# Transcriptomic Diversification of Granulosa Cells during Follicular Development in Chicken

**DOI:** 10.1038/s41598-019-41132-1

**Published:** 2019-04-02

**Authors:** Guoqiang Zhu, Chao Fang, Jing Li, Chunheng Mo, Yajun Wang, Juan Li

**Affiliations:** Key Laboratory of Bio-resources and Eco-environment of Ministry of Education, College of Life Sciences, Sichuan University, Chengdu, 610065 P. R. China

## Abstract

Granulosa cells play important roles in ovarian follicular development. To better understand the molecular mechanisms involved in this physiological process in chicken, high-throughput transcriptome analyses were performed to study the expression profiles of granulosa cells harvested from 6 mm white follicles, F5 follicles and F1 follicles. The analyses elucidated a clear tendency of granulosa cells in shifting its expression profile from proliferation to differentiation during follicular development. Transcripts down-regulated during this process were mainly associated with cell division, cell cycle and DNA replication while the up-regulated transcripts were related to ribosomal function, lipid metabolism and protein synthesis. Our study for the first time provides the complete gene expression profiles along follicular development supporting the active involvement of many genes characterized in cell signaling (*AMH, Inhibins, Activins, BMPs*) and transcription factors (*SMAD3, SMAD5, ID1, ID2, ID3*). Their temporal expression profiles support the notion of continual cross-talk between granulosa cells and its neighboring cells and shed light on the mechanisms behind avian follicular selection and pave the way to the better understanding of reproductive efficiency.

## Introduction

Chicken ovarian follicle contains three types of cells, namely oocyte, granulosa cells and theca cells. Granulosa cells, which form a layer surrounding the oocyte, are the main coordinator for follicular development^1,2^. Granulosa cells synthesize estradiol and progesterone in response to the stimulation of gonadotropins (FSH and LH) respectively, and coordinate follicular maturation and oviposition^3^. On the other hand, granulosa cell-derived Kit ligand (KL) stimulates oocyte growth and inhibits oocyte-derived BMP-15 expression in an orchestrated manner^4^. A recent study reported that follicular selection among the cohort of 8–13 prehierarchal follicles (6–8 mm in diameter) is dependent on the up-expression of FSH receptor (FSHR) transcripts in its granulosa cells, substantiating the key role of granulosa cells in follicular selection^5^.

Within the last few years, high-throughput transcriptome analysis has become a popular and powerful tool to discover novel genes and delineate the complete gene interaction network and has been applied on studies on follicular development. In cows, the transcriptome profile has been analyzed in granulosa cells to study the mechanisms behind dominant follicle selection^6^. The gene cyclin D2 (CCND2) has been implicated in the regulation of granulosa cell proliferation^6–8^. A recent transcriptome study on the bovine granulosa cells reveal the novel biomarkers of follicular status after FSH decline or withdrawal^9^. In pigs, the gene expression profiles of granulosa cell harvested from terminal follicles have been analyzed revealing the involvement of novel genes originally thought to be associated with mainly immune response and inflammation^10^. In horses, the transcriptome analyses reveal the distinct expression profile in granulosa and theca cells from developmental follicles^11^.

In contrast to its wide application in mammals, the use of high-throughput transcriptome analysis in avian models is limited. So far, studies have mainly used microarray analyses to uncover the genes involved in oocyte maturation and early embryonic development. In broiler breeder ovaries, candidate genes involved in ovarian functions and follicle number decision have been identified, for instance, platelet-derived growth factor receptor-like (PDGFRL) which is believed to play a part in steroid-based feedback^12^. Another transcriptome analyses on small yellow follicles has highlighted the potential roles of WNT4 in follicular selection^13^.

The domestic hen ovary is described as a cohort of multiple ovarian follicles, including thousands of primordial follicles, multiple primary follicles (<6 mm), prehierarchal (6–8 mm) and preovulatory follicles (10–40 mm), where in the 3 later stage follicles granulosa and theca cells are enclosed. These ovarian follicles varying in distinct size and developmental stage are excellent research materials for the study of follicular growth, selection and maturation^14^. In the present study, high-throughput transcriptome analyses were employed to study the differential gene expression profile of chicken granulosa cells in three different stages of developmental follicles, aiming to reveal the active roles of these cells during this significant process often associated with reproductive efficiency. Besides, comparison between our current chicken granulosa cell gene expression profiles and previous bovine studies^15^ will also pave the way to the better understanding of vertebrate follicular development.

## Materials and Methods

### Ethics statement

All experiments were performed according to the regulations and guidelines established by the Ministry of Science and Technology of the People’s Republic of China (Approval number: 2006–398). All animal handling procedures followed the animal welfare recommendations and were approved by the Animal Ethics Committee of Sichuan University.

### Animal tissues

**Figure 1:**
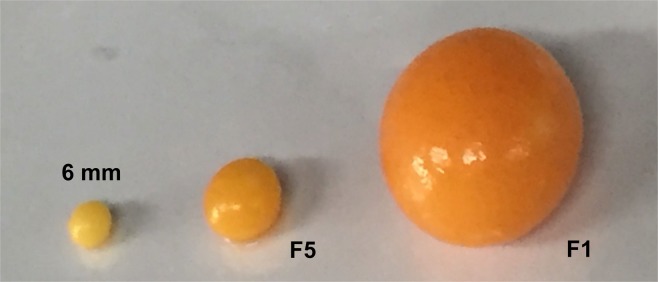
Follicles used for high-throughput RNA analyses. 6 mm: white follicle, or known as prehierarchical follicles prior to the follicular selection; F5, small yellow follicle, also known as early preovulatory follicles after follicular recruitment; F1, the largest yellow follicle, also known as late preovulatory follicles prior to ovulation.

Laying hens with normal follicular hierarchies (Lohmann Layer strain) were kindly provided by MUXING company in Sichuan, China. In this study, follicles were collected from a total of eight hens (n = 8). In general, the hen ovary in peak laying period contains 30–100 small yolky follicles and 4–7 follicles recruited in the hierarchy. Typically, it takes the follicles in average 2 days to grow from 5 mm size to 8 mm size, and another 6 days to develop from 8 mm to pre-ovulatory stage^16^. Due to the time limit and sequencing cost, only follicles of three developmental stages, namely 6 mm, F5 and F1, were selected for the collection of granulosa cells for further analysis based on the characteristics described as follows (Fig. 1). The 6 mm follicles are white follicles that have yet been selected into the hierarchy^17^. The F5 follicles are small yellow follicles with diameter ranging from 11 to 12 mm, and upon recruitment into the hierarchy, their granulosa cells are generally believed to enter into an actively proliferative stage in order to accommodate an increasing deposition of egg yolk precursors in the follicles^18^. The F1 follicles represent the largest follicle found in the hierarchy just before ovulation, with diameter ranging from 38 to 42 mm.

### RNA extraction

Each follicle was subjected to RNA extraction independently. The follicle was soaked first in 1 × PBS solution buffer at 4 °C. Based on the established protocols^4,19^, the granulosa cells were isolated as completely as possible from the rest of the follicular compartment and dispersed in RNAzol reagent (Molecular Research Center, Cincinnati, OH, USA). According to the manufacturer’s instructions, the RNA samples were extracted and then dissolved in diethylpyrocarbonate-treated H_2_O for quality and quantity evaluation.

### RNA-seq library construction and sequencing

RNA-seq libraries were prepared following the standard Illumina protocols by Novogene (Beijing, China). In brief, mRNA (at least 3 μg equally contributed from 8 individuals) were enriched from total RNA by poly-A oligo-attached magnetic beads with an integrity value >8.0. Double-stranded complementary DNAs were synthesized with random hexamer primers and purified with AMPure XP beads. Inserts with expected size were concentrated and subjected for transcriptome analyses.

### Differentially expressed genes

Using Tophat v2.0.12 as a mapping tool^20^, clean reads were filtered from raw reads and mapped to the reference genome of Gallus gallus available on ftp://ftp.ensembl.org/pub/release-72/fasta/gallus_gallus/dna. HTSeq v0.6.1 was used to count the reads mapped to each gene. Typically, both FPKM (Fragment reads Per Kilobase per Million mapped reads) and RPKM (Reads Per Kilobase per Million mapped reads) can be employed for the evaluation of the transcripts abundance. Based on the sequencing depth and gene length for the reads count, FPKM was chosen in the present study as the key parameter in gene expression analyses. A q-value of <0.05, which is adjusted from p-value by the Benjamini& Hochberg method, was employed in this study and the 2-fold minimum differential expression was designated as the threshold of differentially expressed genes^21^.

### Functional gene annotation

Gene ontology (GO) and Kyoto Encyclopedia of Genes and Genomes (KEGG) enrichment analyses were performed by GOSeq Release2.12 and KOBAS v2.0 respectively^22,23^. As mentioned above, terms with q-value <0.05 were considered significantly enriched by differential expressed genes. An online database resource search tool for the retrieval of interacting Genes/Proteins (STRING, http://string-db.org/) was employed for the protein-protein interaction analyses. String score higher than 700 was collected and then subjected for the analyses in software Cytoscape v3.3.0^24^.

### Reverse transcription and quantitative real-time PCR validation

Oligodeoxythymide (0.5 μg) and total RNA (2 μg) were mixed in a total volume of 5 μL, and incubated at 70 °C for 10 mins, then cooled at 4 °C for 2 mins. 1 μL single strand buffer, 0.5 μL each deoxynucleotide triphosphate, 0.5 μg oligo-deoxythymidine, and 100U moloney murine leukemia virus (MMLV) reverse transcriptase (Promega, Madison, WI, USA) were then added into the reaction mix in a total volume of 10 μL. Reverse transcription (RT) was performed at 42 °C for 90 mins.

To further validate the mRNA level of the many genes including *StAR, LHCGR, CYP11A1, CYP51A1, CYP1B1, INHBB, WNT4, SMAD3, SMAD5, ID1, ID2* and *ID3*, quantitative real-time PCR was performed. Primers used for the amplification of the target genes were listed in Table 1. According to our previously established method^25^, the real-time PCR was conducted on the CFX96 real-time PCR Detection System (Bio-Rad, Hercules, CA, USA) in a total volume of 20 μL containing 13.2 μL Mili-Q water, 0.5 μL DMSO, 2 μL RT product, 1 μL single PCR buffer, 0.4 μL 2 mM each dNTP, 0.3 μL 20 mM primer, 0.3 μL Taq DNA polymerase (Invitrogen, Carlsbad, CA, USA), and 1 μL EvaGreen (Biotium Inc., Hayward, CA, USA). The specificity of PCR amplification was first checked by agarose gel electrophoresis and the identity of all PCR products were confirmed by sequencing. The mRNA level of the target genes was first normalized as the ratio to that of *GAPDH* and *EF1A*, and then expressed as the fold difference to the control group. The qPCR data of three groups (n = 8 each) was analyzed by the comparative Ct method^26^, and tested for normality using Student’s t test or by one-way ANOVA followed by the Dunnett test using GraphPad Prism 6.01 (GraphPad Software, San Diego, CA). To validate the results, all experiments were repeated at least twice.

**Table 1:** Primers used for quantitative real-time PCR validation.

Gene	Sense/antisense	Primer sequences	Size(bp)
StAR	sense	CGCTGCCATCTCCTACCAACAC	196
	antisense	GGACATCTCCATCTCGCTGAAGG	
LHCGR	sense	TCAGGCGGATACACAACGATGC	155
	antisense	AGGCGGCAGTCTCTTCAGTG	
CYP11A1	sense	GCCAGCGTCACCGAGATGAT	211
	antisense	GCAGCCTGAGAGTCTCCTTGATG	
CYP51A1	sense	GGTTATCCAGAAGCGTCGGAGTT	133
	antisense	CAGGAGCAGCCCAATGAGCAT	
CYP1B1	sense	GCTGCTCTGGCTCCTCATCTTC	93
	antisense	AGGCGGTCTCTCCCAACGAT	
INHBB	sense	CCGCCTCGCTGATAACAAACAC	138
	antisense	CCTGATGGTGCTATGATCCAGTCA	
WNT4	sense	ACGGAACCTGGAGGTGATGGA	183
	antisense	CCTGCTGAAGAGATGGCGTAGAC	
SMAD3	sense	TGAACGCTTCTGCCTTGGTCTG	90
	antisense	AGCCTCACTCCTCTTCCGATGT	
SMAD5	sense	TGAACGCTTCTGCCTTGGTCTG	209
	antisense	GGCACTAACACTGGAGGTAGAACTG	
ID1	sense	GTGATCGACTACATCTGGGACCTG	127
	antisense	CTCTCTCAGCGGCACAGTATGC	
ID2	sense	ACCACGCTCAACACAGACATCAG	83
	antisense	GCTTTGCTGTCACTCGCCATTAG	
ID3	sense	CAAGCTGAGCCAGGTGGAGATC	195
	antisense	TGATGGAGGAGGCGTTAGTGACA	
GAPDH	sense	TGCTGCCCAGAACATCATCC	199
	antisense	ATCAGCAGCAGCCTTCACTACC	
EF1A	sense	AGCAGACTTTGTGACCTTGCC	90
	antisense	TGACATGAGACAGACGGTTGC	

## Results

### Differential Gene Expression Profiles of Granulosa Cells collected from 6 mm and F5 Follicles

In present study, granulosa cells collected from follicles of the three developmental stages, ie 6 mm, F5 and F1 follicles, were subjected to high-throughput RNA analyses (Fig. 1). All sequencing data were deposited in NCBI public database (accession number: GSE112470). The 6 mm follicles and F5 follicles represent the stages before and after follicular selection, thus their expression profiles were compared in this study, in hope to provide some clues to the molecular mechanisms behind the key process of follicular selection.

As shown in Fig. S1, in a total of 963 transcripts showed differential expression profile between granulosa cells harvested from 6 mm follicles and F5 follicles. Among these, 529 transcripts showed up-regulation while the other 434 transcripts showed down-regulation in the stage of F5 follicles when compared with 6 mm follicles. The top 50 up-regulated genes and the top 50 down-regulated genes were listed in Table 2. Multiple genes actively involved in the follicular development showed great variation in their expression level. For example, the gene *NR5A2* (or the liver receptor homolog 1, *LRH-1*), which is a member of the nuclear hormone receptor superfamily and implicated in various physiological processes from bile acid metabolism to steroidogenesis^27^, showed a significant increase in expression with a Log_2_ (fold change) value of 9.08. Elevated expression was also observed in *RASD1* gene, Ras-related dexamethasone-induced 1 protein [Log_2_ (fold change) value = 4.19]. *Transgelin*, which encodes the actin cross-linking protein and has been described as an early and sensitive marker for the onset of transformation^28^, showed the greatest reduction in expression with Log_2_ (fold change) value of 11.27. *COL3A1* transcript, which encodes the protein found in connective tissues and involved in cell-matrix interactions^29^, also showed significant down-regulation with Log_2_ (fold change) value of 7.70.

**Table 2:** Top 50 genes showing significant up or down regulation in granulosa cells collected from 6 mm and F5 follicles. (q < 0.05).

Gene name/Gene_ID	Fold change (Log_2_)	Gene name/Gene_ID	Fold change (Log_2_)	Gene name/Gene_ID	Fold change (Log_2_)
**Up-regulated transcripts - higher in granulosa cells in F5 than in 6 mm follicles**					
*CEL*	10.15	ENSGALG00000010364	5.77	*EMP1*	4.58
*NR5A2*	9.08	*CYP11A1*	5.74	*MAP2*	4.57
*BPIFB3*	8.10	*TSPAN1*	5.65	ENSGALG00000004692	4.55
ENSGALG00000028815	7.83	ENSGALG00000010303	5.56	*SESN2*	4.54
ENSGALG00000004721	7.77	*INF2*	5.45	*P2RX2*	4.47
*SPTSSB*	7.72	*FLVCR2*	5.36	ENSGALG00000019077	4.45
*KCNH2*	7.38	*SORL1*	5.35	ENSGALG00000025791	4.40
*TMEM72*	7.16	*INHA*	5.30	*PPARGC1B*	4.38
*ABHD3*	7.06	*ATP8A1*	5.23	*ELAVL4*	4.36
*MFSD2B*	6.63	*KREMEN1*	5.19	*ESRRB*	4.35
*TYRP1*	6.42	*PLEKHA6*	5.05	*DMRT2*	4.32
*PGF*	6.39	*LOXL1*	5.05	*KCNAB1*	4.31
ENSGALG00000010018	6.21	*PLCG1*	5.01	*STRA6*	4.25
*FABP5*	6.18	*ZPD*	4.97	*RHBDL3*	4.21
*SRL*	6.16	*STAR*	4.85	*PPAR*	4.20
*TNNI1*	6.03	*PRDM77*	4.75	*RASD1*	4.19
ENSGALG00000008936	5.78	*LRRN4*	4.69		
**Down-regulated transcripts - lower in granulosa cells in F5 than in 6 mm follicles**					
*TAGLN*	11.27	*COL6A2*	7.07	ENSGALG00000026616	5.76
*OSF-2*	9.37	*HSPB1*	6.88	*SERPINB2*	5.71
*GIF*	9.13	*AQP1*	6.81	*THY1*	5.52
*COL6A1*	8.64	*TGM2*	6.61	*KRT14*	5.46
*TGM4*	8.55	*MMP9*	6.41	*GLUL*	5.41
Novel00694	8.55	*COL12A1*	6.35	*HTRA1*	5.33
*DES*	8.45	*ANPEP*	6.35	*FXYD6*	5.26
*F13A1*	8.44	*DCN*	6.33	*EDN2*	5.10
*PTRF*	8.43	*COL6A3*	6.26	ENSGALG00000005956	5.08
*CCDC80*	8.21	*HPGDS*	6.24	*CLEC3B*	5.07
*COL16A1*	7.81	*TCF21*	6.17	*IGJ*	5.06
*IQCA1*	7.80	*ANGPT4*	6.03	*FBLN1*	5.05
*COL3A1*	7.70	*CALD1*	5.97	ENSGALG00000016556	5.04
*ZP1*	7.68	*MGP*	5.86	*ALDH6*	4.85
ENSGALG00000023973	7.39	*CFD*	5.85	*KRT8*	4.83
*PPL*	7.36	ENSGALG00000010250	5.85	*SOD3*	4.81
ENSGALG00000021139	7.21	*FBLN2*	5.77		

Besides these genes with significant variation in their expression, numerous genes with increased expression were identified, which can be grouped under similar functions as below: cholesterol metabolism (*StAR, CYP11A1* and *SORL1*), potassium ion transmembrane transport (*KCNAB1, KCNJ12, KCNJ5* and *SLC8A5*), dendrite morphogenesis (*ELAVL4* and *MAP2*), steroid hormone receptor activity (*ESRRB, NR5A2* and *PPARG*) and carboxylic ester hydrolase activity (*AADACL2* and *CEL*). In contrast, the genes demonstrating a reduced expression are mainly implicated in peptide cross-linking (*F13A1, TGM2* and *TGM4*), negative regulation of protein kinase activity (*THY1, DCN* and *HSPB1*), extracellular matrix organization (*FBLN1, MMP9* and *POSTN*) and angiogenesis (*THY1, ANGPT4* and *CCDC80*).

**Figure 2:**
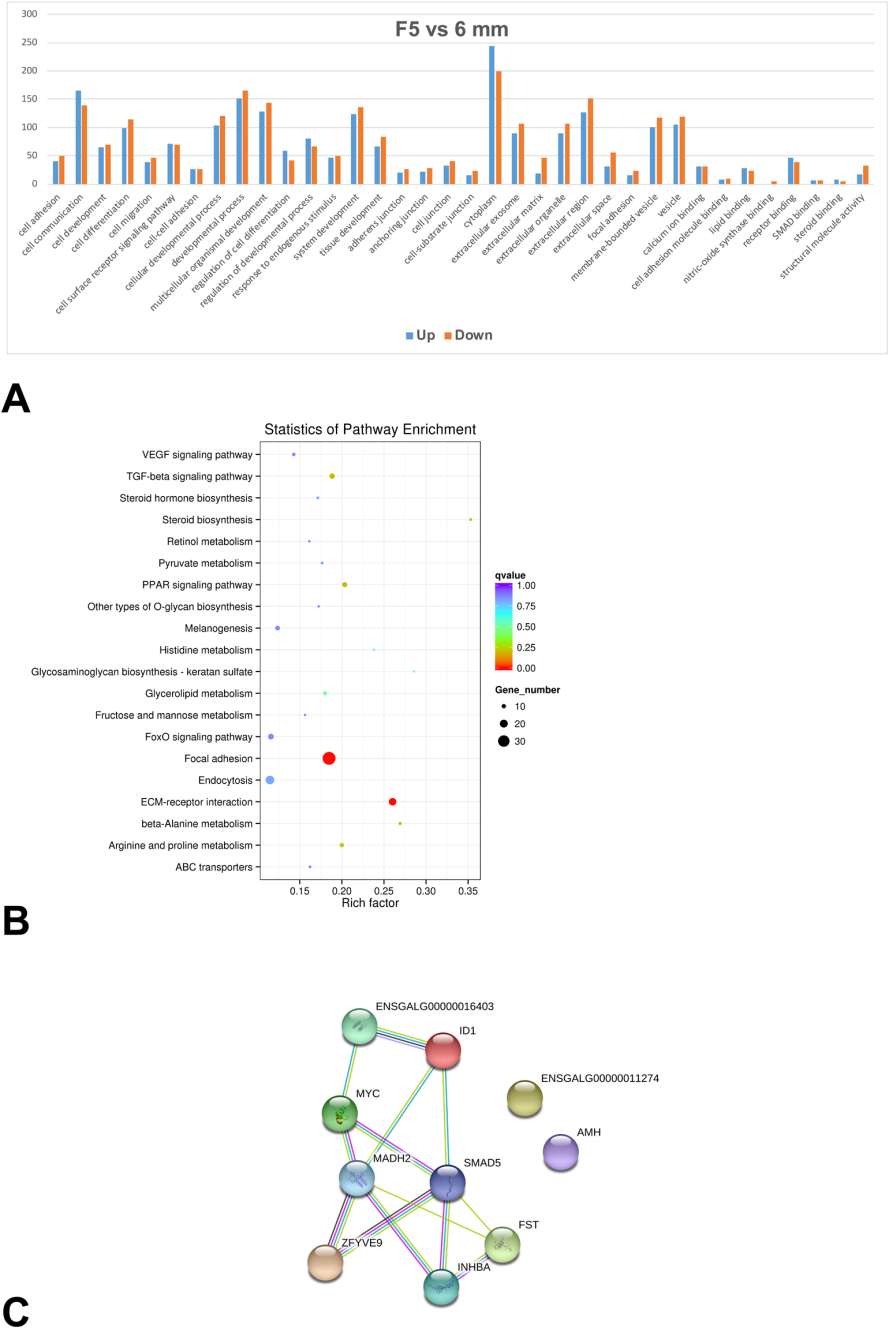
Gene expression profiles of granulosa cells from 6 mm follilcles and F5 follicles. (**A**) Gene ontology (GO) functional enrichment of genes differentially expressed in granulosa cells collected from 6 mm and F5 follicles. The y-axis and x-axis indicate the number of genes in each cluster and the names of clusters respectively. (**B**) Scatter plot of enriched KEGG pathways for differentially expressed genes in granulosa cells collected from 6 mm and F5 follicles. The rich factor is the ratio of the differentially expressed gene number to the total gene number in a certain pathway. The size and color of the dots represent the gene number and the range of the q-value, respectively. (**C**) The TGF-beta signaling pathway network identified in granulosa cells collected from 6 mm and F5 follicles based on STRING database.

In an effort to reveal the relationship between these genes and known biological processes, cellular components and molecular functions, the GO terms of the significantly enrichment of differentially expressed transcripts were summarized. As shown in Fig. 2A, 216 GO terms were categorized into various biological processes including cell adhesion (GO: 0007155), cell communication (GO: 0007154), cell differentiation (GO: 0030154) and system development with 259 genes (GO: 0048731). On the other hand, a total of 36 GO terms were categorized into various cellular components including anchoring junction (GO: 0070161), cell junction (GO: 0030054), extracellular matrix (GO: 0031012) and focal adhesion with 39 genes (GO: 0005925). A total of 17 GO terms were categorized into molecular function including calcium ion binding (GO: 0005509), receptor binding (GO: 0005102), SMAD binding (GO: 0046332) and steroid binding with 13 genes (GO: 0005496).

KEGG pathway analysis was also performed to investigate how these differentially expressed genes were involved in different cellular processes. As shown in Fig. 2B, with the threshold of q-value < 0.05, the large dots clearly showed the genes were mainly related to pathways such as focal adhesion, extracellular matrix (ECM)-receptor interaction and endocytosis. Besides pathways mentioned above, these genes also matched to other pathways including steroid biosynthesis, FoxO signaling pathway, PPAR signaling pathway, TGF-beta signaling pathway, arginine and proline metabolism, glycerolipid metabolism and histidine metabolism. To reveal how these genes may interact, protein-protein interaction analyses were carried out based on the STRING database. In particular, 10 genes identified in the present study were found to be associated with TGF-beta signaling pathway (gga04350) (Fig. 2C).

### Differential Gene Expression Profiles of Granulosa Cell in F5 and F1 Follicles

The transcriptomes of granulosa cells harvested from F5 and F1 follicles were analyzed in comparison, aiming to reveal the differential gene expression profiles along the large follicle development.

As shown in Fig. S2, 1239 transcripts showed differential expression profile between granulosa cells collected from F5 and F1 follicles. Among them, 529 transcripts showed elevated level in the more developed F1 follicle when compared with F5 follicle, while the remaining 710 transcripts showed reduced level. The top 50 up-regulated genes and top 50 down-regulated genes were presented in Table 3. Some genes showed the continual up-regulation tendency starting from 6 mm follicles. For example, *RASD1* transcript showed up-regulation with Log_2_ (fold change) value of 5.24. Similarly, *STAT6* transcript, which plays a central role in the modulation of anti-apoptotic activity of IL4^30^, showed a significant increase with Log_2_ (fold change) value of 4.72. In contrast, *SMAD7a* transcript, which is I-SMAD family member and known to compete with SMAD4 and negatively regulate TGF-beta signaling^31^, showed a substantial down-regulation with Log_2_ (fold change) value of 9.16. Similar reduction in expression level is also observed in *EDN1* transcript with Log_2_ (fold change) value of 5.63.

**Table 3:** Top 50 genes showing significant up or down regulation in granulosa cells from F5 and F1 follicles. (q < 0.05).

Gene name/Gene_ID	Fold change Log_2_	Gene name/Gene_ID	Fold change Log_2_	Gene name/Gene_ID	Fold change Log_2_
**Up-regulated transcripts-higher in granulosa cells in F1 than in F5 follicles**					
ENSGALG00000028221	8.61	ENSGALG00000006644	5.36	*RRP36*	4.78
*KIAA1211*	7.58	*ENTPD2*	5.33	*VWC2*	4.77
*EPAS1*	7.51	*TRIM14*	5.30	*STAT6*	4.76
ENSGALG00000002771	7.36	*PRSS35*	5.27	*WSCD2*	4.68
*HTRA1*	7.28	*RASD1*	5.24	*GREM1*	4.66
*KCNJ5*	7.08	*TNR*	5.21	*PIP5K1B*	4.64
*NIM1*	6.66	*CYP1B1*	5.18	*RELT*	4.64
*THSD7A*	6.28	*ADGRD2*	5.06	*KIAA1217*	4.56
*RPH3A*	6.00	ENSGALG00000027201	5.03	*PTCH2*	4.52
*SYNPR*	5.95	ENSGALG00000000038	4.99	*ADAMTS3*	4.49
*LHCGR*	5.83	*CDH4*	4.97	*CTNNA2*	4.49
*KNDC1*	5.65	*KIAA0040*	4.96	*APOH*	4.48
*KLHL32*	5.63	*ATRNL1*	4.88	*PDLIM5*	4.47
*ADCYAP1R1*	5.53	*PDE4D*	4.83	*FADS6*	4.41
*SYNDIG1L*	5.47	*EFCC1*	4.81	Novel00150	4.38
*IGSF10*	5.45	*CLDN5*	4.81	*SLCO3A1*	4.27
*ACSS1*	5.37	*STK17A*	4.78		
**Down-regulated transcripts-lower in granulosa cells in F1 than in F5 follicles**					
SMAD7a	9.16	ENSGALG00000023172	6.06	*ADRA2A*	5.03
*INHBB*	8.40	*ITGA11*	6.05	*FST*	5.00
*SLCO2B1*	8.38	*RGS5*	5.95	*NOXO1*	5.00
*RSPO3*	7.88	*SPTSSB*	5.93	*POLQ*	4.97
*MMEL1*	7.82	*PHYHD1*	5.84	*COL11A1*	4.95
*FABP4*	7.55	*NEURL1B*	5.78	*SLC30A2*	4.95
*TSPAN1*	7.37	*DPYSL3*	5.69	*WBSCR17*	4.95
*COL5A1*	7.30	ENSGALG00000020819	5.65	*FBN1*	4.79
*COL4A1*	7.20	*ZP2*	5.63	*NEXN*	4.79
*NLGN3*	6.77	*EDN1*	5.63	*PRDM16*	4.77
*FABP5*	6.61	*ADAM11*	5.63	*SVEP1*	4.75
*IL1RL1*	6.54	*CREB3L1*	5.29	*BPIFB3*	4.73
*LTBP1*	6.49	*GPRC5C*	5.22	*CTHRC1*	4.71
*WNT4*	6.44	*LOXL2*	5.19	*SIDT1*	4.69
*ARHGAP8*	6.32	*TNNI1*	5.17	*MPZL2*	4.68
*SLC15A1*	6.17	*EMP1*	5.14	*TMEM72*	4.66
*CEL*	6.15	ENSGALG00000020719	5.08		

Besides genes mentioned above, up-regulation was shown in genes associated with the negative regulation of BMP signaling pathway (*GREM1* and *VWC2*), uterus development (*ESR1* and *LHCGR*) and heart rate regulation (*EPAS1* and *PDE4D*). In contrast, down-regulation was noted in genes related to collagen fibril organization (*COL5A1, COL11A1* and *LOXL2*), response to hypoxia (*EGLN3, EDN1* and *LOXL2*), modulation of synaptic transmission (*CEL* and *NLGN3*) and glucose transport (*EDN1* and *FABP5*) and extracellular matrix (*OCX36*, *FBN1* and ZP2).

**Figure 3:**
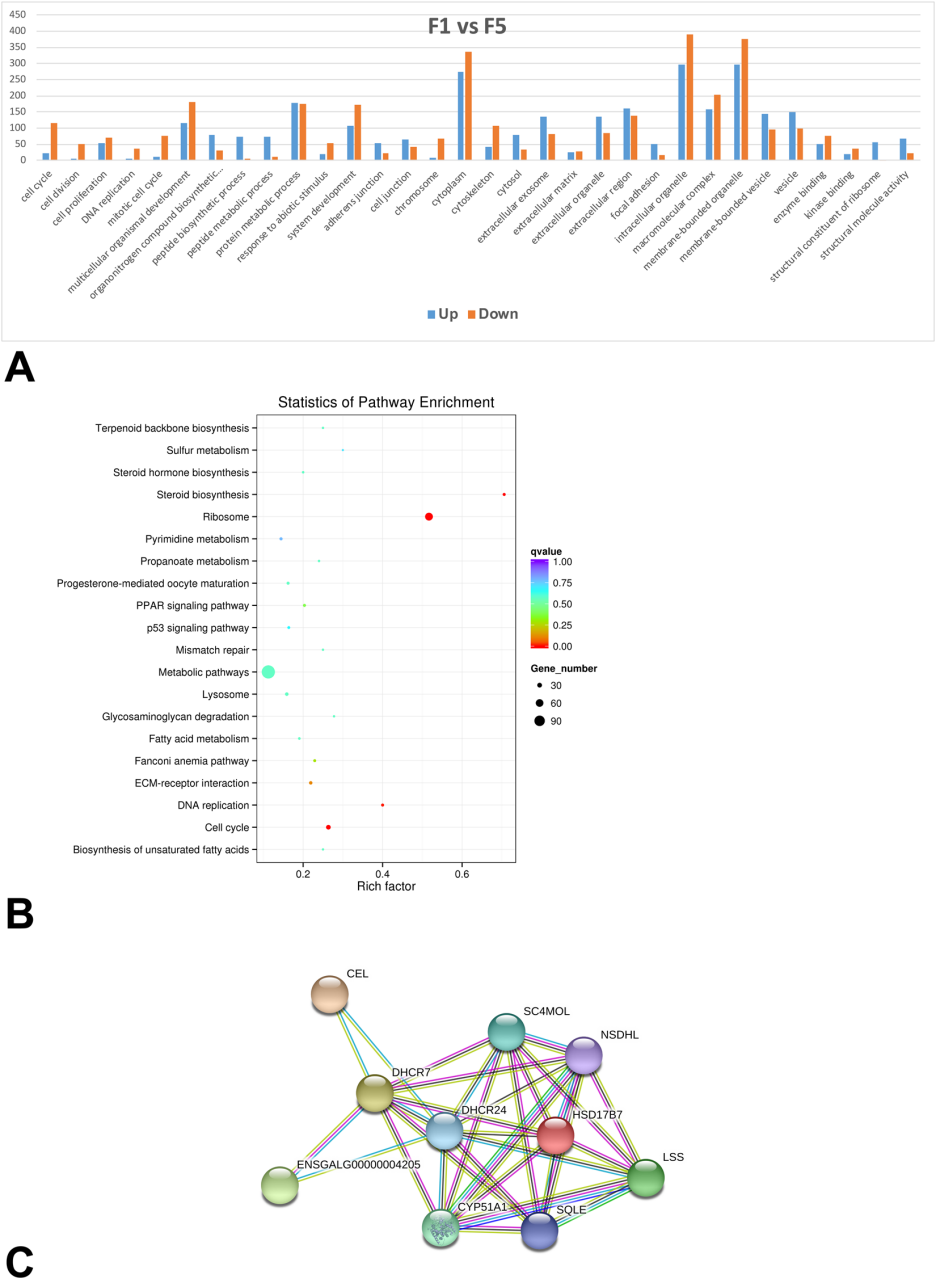
Gene expression profiles of granulosa cells collected from F5 and F1 follicles. (**A**) Gene ontology (GO) functional enrichment of genes differentially expressed in granulosa cells collected from F5 and F1 follicles. The y-axis and x-axis indicate the number of genes in each cluster and the names of clusters respectively. (**B**) Scatter plot of enriched KEGG pathways for differentially expressed genes expressed in granulosa cells collected from F5 and F1 follicles. The rich factor is the ratio of the differentially expressed gene number to the total gene number in a certain pathway. The size and color of the dots represent the gene number and the range of the q value, respectively. (**C**) The steroid biosynthesis network identified in granulosa cells collected from F5 and F1 follicles based on STRING database.

To reveal the functional aspects of these differentially expressed genes, their GO terms are summarized in Fig. 3A. 281 genes were mapped to 72 GO terms categorized in biological processes including the cell cycle (GO: 0007049), cell division (GO: 0051301), cell proliferation (GO: 0008283) and system development (GO: 0048731). On the other hand, 150 genes were mapped to 67 GO terms categorized in the following cellular components, namely the cell junction (GO: 0030054), cell surface (GO: 0009986), extracellular matrix (GO: 0031012) and cytoskeleton (GO: 0005856). And 89 genes were matched to 4 GO terms categorized in the following molecular function, namely enzyme binding (GO: 0019899), kinase binding (GO: 0019900), structural constituent of ribosome (GO: 0003735) and structural molecule activity (GO: 0005198).

KEGG pathway analysis of these differentially expressed genes was shown in Fig. 3B. Using threshold q-value of <0.05, majority of these genes were associated with pathways such as ribosome function, steroid biosynthesis, cell cycle and DNA replication. Besides these 3 major pathways, other pathways such as ECM-receptor interaction, fanconi anemia pathway, PPAR signaling pathway, lysosome, glycosaminoglycan degradation, metabolic pathways and propanoate metabolism were also identified in our study. Protein-protein interaction analyses based on the STRING database showed 11 of these genes playing role in steroid biosynthesis (gga00100) (Fig. 3C).

### Differential Gene Expression Profiles between Chicken and Bovine Granulosa Cells

Vertebrate ovarian follicles share similar developmental stages including primordial follicle initiation, growth, selection, maturation and the final stage, ovulation^32,33^. In cows, granulosa cells collected from small follicles prior to follicular selection (size <5 mm) and large follicles prior to ovulation (size >10 mm) have been analyzed in previous studies^15^. In the present study, the developmental stages (prior selection: 6 mm follicles, prior ovulation: F1 follicles) of chicken follicles selected in this study were well in corresponding with the follicles selected in bovine, thus their gene expression profiles were compared which provided us the unique opportunity to reveal the intricate mechanism on follicular development across species.

**Figure 4:**
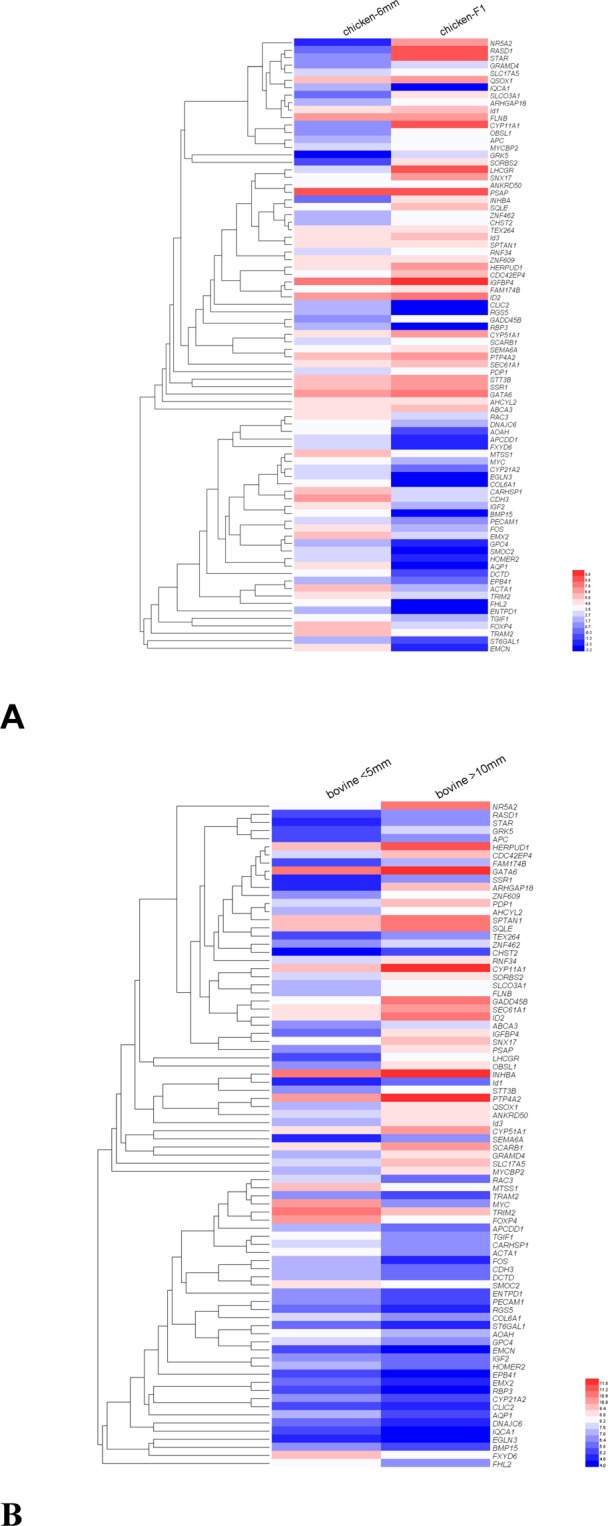
Heatmap displaying the gene expression profiles of granulosa cells in (**A**) chicken and (**B**) bovine. The FPKM values were extracted from the chicken RNA-seq data in present study and the bovine Affymetrix microarrays data (NO:GSE39589). 82 genes were identified to show similar expression pattern in granulosa cells in chicken and bovine follicles.

As shown in Fig. 4, the hierarchical clustering analyses resulted in two differential expression gene clusters between granulosa cells harvested from the two follicular stages which is conserved between bovine and chicken transcriptome findings. Among these, 46 genes showed up-regulation (Fig. 4A) and 36 genes showed down-regulation (Fig. 4B). The FPKM values of these genes from chicken and bovine studies were listed in Table S1.

As shown in Table S1, genes involved in steroid synthesis (*NR5A2, RASD1, StAR, CYP11A1, CYP51A1, SCARB1, LHCGR, CYP21A2*) were found to show similar expression pattern between the species. Genes associated with intracellular enzymes, protein synthesis and transportation (*SORBS2, HERPUD1, SQLE, SEC61A1, SSR1, QSOX1, TRAM2, AOAH, DCTD, EGLN3*), and genes encoding the key intracellular signaling proteins (*GRK5, SNX17, ARHGAP18, APC, OBSL1, GADD45B, RNF34, PDP1, CHST2, GRAMD4, PTP4A2, STT3B, AHCYL2, PSAP, RAC3, APCDD1, CARHSP1, RGS5, IQCA1, DNAJC6, HOMER2, MTSS1, SMOC2*) were noted in the comparison. In addition, genes encoding cytoskeleton constituents and the extracellular matrix components (*CDC42EP4, SPTAN1, FLNB, TEX264, EPB41, CDH3, ACTA1, FHL2, EMCN, COL6A1*), genes encoding transmembrane transporters, channel proteins and receptors (*SLCO3A1, SEMA6A, SLC17A5, ABCA3, FAM174B, ST6GAL1, GPC4, CLIC2, ENTPD1, FXYD6, AQP1, RBP3, PECAM1*), genes encoding transcription factors (*IDs, GATA6, ZNF462, ZNF609, ANKRD50, MYCBP2, TGIF1, EMX2, TRIM2, FOS, FOXP4, MYC*) and genes encoding the signaling molecules (*INHBA, IGFBP4, IGF2, BMP15*) also shared similar expression profile between the species in two developmental stages.

### Validation of gene expression profiles

**Figure 5:**
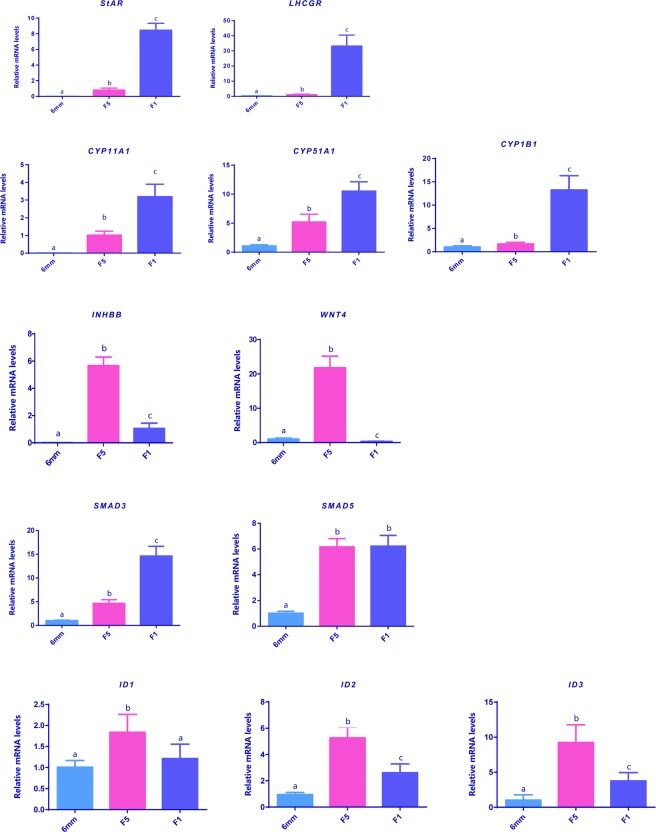
Quantitative real-time PCR validation of differentially expressed genes identified in transcriptome sequencing. The results were normalized based on the housekeeping genes *GAPDH* and *EF1A*.

To confirm the gene expression profiles obtained from the high-throughput RNA sequencing, quantitative real-time PCR analyses were also performed in the present study. Genes showing significant differential expression profiles between the 3 developmental stages including those associated with steroid synthesis (*StAR, LHCGR, CYP11A1, CYP51A1, CYP1B1*), signaling molecules (*INHBB, WNT4*), TGF-beta family downstream mediators (*SMAD3, SMAD5*) and transcription factors (*ID1, ID2, ID3*) were subjected to qPCR analyses. As shown in Fig. 5, their expression profiles were in line with the high-throughput sequencing data.

## Discussion

In the present study, high-throughput transcriptome analyses were employed to study the differential gene expression profiles of granulosa cells collected from three different developmental stages. In addition, the expression profiles of granulosa cells from chicken follicles were compared with that in bovine follicles, in the hope to find some clues to better understand the mechanisms behind vertebrate follicular development.

### Comparison of expression profiles between granulosa cells in chicken 6 mm follicles and F5 follicles

In present study, 963 transcripts showed differential expression profiles between granulosa cells harvested from 6 mm follicles and F5 follicles. The 6 mm  follicles showed abundance in transcripts mainly associated with cell cycle, cell division and DNA replication. This finding falls in line with previous reports that hen granulosa cells selected at this stage are mitotically active and undifferentiated^34,35^. In contrast, granulosa cells in F5 follicles were rich in transcripts associated with cell proliferation, cell communication and cell differentiation in general, supporting the phenomena of fast growth with cell differentiation and maturation characteristic in this developmental stage^36^.

The 529 transcripts showing substantial up-regulation are mainly related to cell communication, developmental processes, multicellular organismal development and cytoplasm in granulosa cells when comparing 6 mm and F5 follicles. On the other hand, GO analyses showed that, the 434 down-regulated transcripts, besides being related to the above processes and cellular components, were also found to be associated with extracellular matrix, extracellular organelles and extracellular regions (Fig. 2A). Different from the mammals, the chicken follicles rely on granulosa cells to transport macromolecules into the oocytes due to the lack of the lumen^37^. These down-regulation transcripts associated with extracellular matrix likely reflect the changes in cargo and transportation route in granulosa cells between the two developmental stages. In small white follicles, the lipoprotein-rich white yolk is continually transported through tight junctions^37^. In larger follicles such as F5 follicles, the lipid-rich egg yolk produced from the liver diffuses through the perivitelline membrane to reach the yolk precursor receptor LR8^38^. KEGG analyses in present study also highlighted the focal adhesion and ECM-receptor interaction pathways, supporting the coordination between granulosa cells and its environment in fulfilling the distinct roles they play along follicular development.

Follicular development is an intricate process involving a very refined coordination between different cells, in which granulosa cells undergo cell growth and differentiation under the constant influence of complicated environmental cues. Thus, it is not surprising that 304 differentially expressed transcripts were mapped to the GO category of cell communication in our study. In particular, as shown in the analysis with STRING database (Fig. 2C), the TGF-beta signaling pathway was identified, supporting its active role in follicular development^36^. For example, Activin A and FSH may control tight junctions through their regulation on occludin expression in granulosa cells^37^. The role of TGF-beta signaling molecules in the follicular development will be further discussed in later section.

### Comparison between expression profiles of granulosa cells in chicken F5 follicles and F1 follicles

In the present study, the F1 and the F5 follicles were subjected for the analyses and compared in an effort to reveal the genes involving the large follicle development. In a total of 1239 transcripts showed the differential expression profile. Along the follicle growth, the up-regulated transcripts were mainly associated in peptide biosynthetic processes, peptide metabolic processes and structural constituents of ribosome reflecting the cell’s state of differentiation. Supporting this hypothesis are the elevated levels of genes encoding LHCGR (luteinizing hormone receptor, LHR) and StAR (steroidogenic acute regulatory protein, StAR), which are markers involved in steroid synthesis^39,40^. Their up-regulation was also validated by real-time PCR analyses (Fig. 5A). The cell fate of full differentiation was further supported by results of KEGG analyses, in which 11 genes were identified in steroid biosynthesis (gga00100). Among them are *CYP11A1, CYP51A1, CYP1B1* which encode key enzymes in steroid synthesis^41,42^. On the other hand, in coordination with enhanced transcripts associated with steroidogenesis, the transcripts involved in cytoskeleton showed down-regulation. This likely suggests the reorganization of intracellular infrastructure to accommodate for the need in transport of steroids. In addition, many transcripts were identified with cell cycle, cell division and DNA replication in GO analyses showed down-regulation, which falls in line with the physiology of granulosa cells moving away from proliferation in the final stage of follicular development.

Interestingly, the number of down-regulated transcripts (710) is much higher than that of up-regulated transcripts (529) in this study. The reason for this may be related with the significant down-regulation of many signaling molecules such as WNT4, IGF2 and their intracellular mediators. In chicken, the increase in follicular size, especially upon the increase in deposition of yolk protein into the oocytes, may have limited the efficiency of these signaling molecules which have paracrine/autocrine mode of action and rely mainly on simple diffusion for transfer^36^. Alternatively, steroids and their by-products can diffuse across cell membrane freely thus being efficient (fast) in signal transduction^43^. The differentiation of granulosa cell and their elevated expression of transcript in steroid synthesis may have allowed the cells to shift to a more effective signaling pathway. Thus, along the follicle growth, the signaling molecules-dependent communication between the granulosa cells may decline.

### Comparison between expression profiles of chicken and bovine granulosa cells

Findings in our present study on 6 mm follicles (prior to follicular selection) and F1 follicles (prior to ovulation) in chickens were compared with the bovine findings to uncover the intricate mechanisms on follicular development across species. As shown in Table S1, many transcripts involved in lipid synthesis and metabolism network (*NR5A2, RASD1, StAR, CYP11A1, LHCGR, CYP51A1, SCARB1, CYP21A2*) were found to be conserved between chicken and bovine expression profiles, reflecting their similar differentiation mechanism leading to fully steroidogenic granulosa cells prior to ovulation^15^. In addition, cell morphology will change to be in line with the differentiation. Genes associated with cytoskeletal constituents (*SPTAN1, EPB41, ACTA1*) and genes implicated in their assembly and association with extracellular matrix (*CDC42EP4, FLNB, PECAM1, CDH3, EMCN, COL6A1*) were found to be conserved in chicken and bovine expression profile, supporting that both might experience similar need for a change in morphology^15^. To be coordinated with these changes, genes associated with intracellular signaling (*SORBS2, IGFBP4, SNX17, ARHGAP18, APC, PDP1, CHST2, GRAMD4, PTP4A2, STT3B, AHCYL2, PSAP, RAC3, FHL2, RBP3, RGS5, IQCA1, AOAH, CARHSP1, MYC, TRIM2, DNAJC6, HOMER2, CLIC2, OBSL1, GADD45B, RNF34, MTSS1*), substance transmembrane transportation (*SLCO3A1, SLC17A5, ABCA3, ST6GAL1, GPC4, ENTPD1, FXYD6, AQP1*) and protein synthesis and secretion (*HERPUD1, SEC61A1, SSR1, TRAM2*) showed similar up-regulation in both chicken and bovine follicles. The similar trend observed in these species in steroid synthesis, cytoskeleton adjustment and substrate transportation supporting that vertebrate follicles undergo similar developmental processes^32,33^. In fact, follicular development differs greatly between chicken and bovine. In contrast with granulosa cells lining the antrum in bovine follicles which are responsible for the maintenance of follicular fluid, granulosa cells in chicken engulf the whole oocyte and are responsible for the fast transportation of a large amount of liver-derived yolk protein precursors^18^. The above transcripts showed similar trend but they differed in FPKM value. For example, *INHBA* (*βA)*, as the subunit for activin A (βA-βA), showed a high abundance in bovine granulosa cells but a low abundance in chicken granulosa cells. The difference may result from the different methodology employed with differential sensitivity. On the other hand, the differential abundance of *INHBA* (βA) between species reflect their different strategy pushing follicular development since activin A (βA-βA) is implicated in regulating granulosa cell responsiveness to gonadtropins^44^.

### Expression profiles of the key genes in TGF-beta signaling pathway

TGF-beta superfamily were reported to be actively involved in follicular development^36^. In the present study, protein-protein interaction analyses on the differential expressed transcripts in F5 and 6 mm follicles highlighted 10 genes involved in TGF-beta signaling pathway (gga04350), emphasizing their importance in this process. Thus, several key members in TGF-beta superfamily were further studied in an effort to provide more insights on their potential reaction time and downstream targets (Table 4).

**Table 4:** The FPKM value of transcripts identified in TGF-beta signaling.

Gene Name	Gene_ID	6mm_FPKM	F5_FPKM	F1_FPKM
***Signaling molecules***				
AMH	ENSGALG00000024368	384.3	18.2	0.8
TGFB2	ENSGALG00000009612	4.8	0.7	0.4
TGFB3	ENSGALG00000010346	16.8	22.0	8.8
INHA	ENSGALG00000011234	132.0	4631.3	3013.0
INHBA	ENSGALG00000012327	1.1	9.5	53.3
INHBB	ENSGALG00000028770	321.2	893.1	1.7
FST	ENSGALG00000014908	290.1	30.4	0.6
BMP2	ENSGALG00000008830	2.5	8.5	0.9
BMP4	ENSGALG00000012429	40.5	55.5	38.9
BMP6	ENSGALG00000012787	3.8	0.4	0.1
***Receptors and their interacting proteins***				
BMPR-II	ENSGALG00000008459	76.5	96.8	106.7
DCN	ENSGALG00000011274	38.2	0.0	0.1
***Transcription factors and intracellular regulators***				
SMAD1	ENSGALG00000009977	16.0	16.3	13.4
SMAD2	ENSGALG00000014697	221.5	1701.0	1874.7
SMAD3	ENSGALG00000007870	29.0	33.4	175.8
SMAD5	ENSGALG00000006309	70.9	176.5	202.4
SMAD6	ENSGALG00000025898	7.4	4.9	1.5
SMAD7a	ENSGALG00000018639	204.9	106.7	0.1
SMAD9	ENSGALG00000017050	1.8	0.4	0.4
ID1	ENSGALG00000006210	31.3	115.1	75.2
ID2	ENSGALG00000016403	191.3	424.5	262.1
ID3	ENSGALG00000027300	39.6	126.6	99.7
MYC	ENSGALG00000016308	23.8	7.3	5.1
RBL1	ENSGALG00000001332	4.7	5.0	0.8
SMURF1	ENSGALG00000004655	10.8	15.8	33.9
ZFYVE9	ENSGALG00000010613	18.6	46.5	62.4

As a TGF-beta superfamily member, anti-mullerian hormone (AMH) binds its specific receptor to promote cell proliferation^36^. In female birds, anti-mullerian hormone is known for repressing the development of mullerian duct and the right ovary^45^. In present study, the FPKM value of *AMH* in 6 mm follicle was 384.3, while it showed a reduction in F5 and F1 follicles with FPKM values of 18.2 and 0.8 respectively. This drastic decline in its expression level is in line with previous report of abundant *AMH* expression in small follicles^46^. AMH is implicated in inhibiting the growth and maintaining the resting phase of remaining primordial follicles. In *AMH* knockout mice, female mice are fertile while its ovaries showed a significant number of small growing follicles^47,48^. In chickens, AMH-rich testis-conditioned medium enhances proliferation of granulosa cell in a dose-dependent manner, hinting that AMH may regulate granulosa cells via autocrine/paracrine route^49^. In cultured granulosa cells harvested from bovine small follicles, *AMH* mRNAs are actively up-regulated by BMPs secreted from oocytes^50^. However, treatment of oocyte-conditioned medium inhibited *AMH* mRNA expression in a dose-related manner in chicken granulosa cells^46^. The protein factors other than GDF9 and BMP15 from oocyte are suggested to regulate the mRNA expression of AMH^46^. Since the AMH reduction is reported to relieve its inhibition on FSHR expression, AMH is suggested to play a part in follicular selection^17^. The finding of potential protein factors will help to reveal the mechanism behind AMH actions in follicular selection.

In mammalian antral follicles, TGF-beta isoforms (TGF-Bs) regulate granulosa and theca cell function in a paracrine/autocrine manner^51–53^. In addition, these molecules show variation in their expression levels in different developmental stages and species specific pattern^36^. Although TGF-B1 was not detected in the present study, both *TGF-B2* (FPKM <4.8) and *TGF-B3* (<22.0) showed relatively low level across the three developmental stages studied, suggesting TGF-Bs may not play an important role during avian follicular development.

Belonging to TGF-beta superfamily, inhibin and activin are believed to play important roles in ovarian functions^54,55^. As a hetero-dimer, inhibin is composed of one α-subunit (INHA) and one of two β-subunits, namely βA (INHBA) and βB (INHBB). The increase in inhibin expression may have helped to maintain androgen synthesis in theca cells, which can serve as a source of substrate for estrogen synthesis during the preovulatory phase^56^. In present study, based on the transcriptome analyses, the expression levels of the subunits *INHA*, *INHBA* and *INHBB* were summarized which shed light on their intricate time-frame among developmental follicles. The FPKM value of *INHA* varied from 132 (6 mm follicles), 4631.3 (F5 follicles) to 3013 (F1 follicles) which is in line with previous study using Northern blot^57^. In accordance with the detection for *INHBA* transcript^58^, the FPKM value of *INHBA* is low, varying from 1.1 (6 mm follicles), 9.5 (F5 follicles) to 53.3 (F1 follicles). Thus, the abundance of inhibin-A (consisted of α/βA subunits) is predicted to be low across the developmental stages with the highest level in the largest follicles. In present study, the FPKM value of the *INHBB* varied from 321.2 (6 mm follicles), 893.1 (F5 follicles) to 1.7 (F1 follicles), hinting that inhibin-B (consisted of α/βB subunits) may concentrate in F5 follicles, being consistent with the immunoreactive assay^59^. Taken together, temporal expression of inhibin-A and inhibin-B differ along follicular development.

Activin is a homo-dimer composed of two β  subunits (Activin-A: βA/βA, Activin-AB: βA/βB, Activin-B: βB/βB). So, the low level of *INHBA* mentioned earlier also suggests a low expression of activin-A (βA/βA) across the developmental stages. Activin-A stimulates the expression of FSHR and LHR in large follicles, and is suggested to play an important role in regulating the responsiveness of granulosa cells to gonadotropins^44^. The relatively low level of activin-A found in the present study supports a former notion that the activin-A from neighboring source other than granulosa cells may participate in this process^60^. As previously mentioned, *INHBB* transcript showed the highest expression level in F5 follicles, which is in line with the observation that the expression of βB-mRNA was not detected in the 4 largest follicles (F4-F1)^61^. In addition, in small yellow follicles, activin-B and FSHR were found to be co-expressed in abundance, and proposed for their role in follicular selection^61^. However, activin-B showed minimal effect on *LHR* and *FSHR* expression in cultured avian granulosa cells^61^. Thus, activin-B might play a role in proliferation of avian granulosa cells to coordinate the fast growth of the follicle and incorporation of large amount of yolk protein precursors into the oocyte. As TGF-beta superfamily members, activins and inhibins bind their receptors depending on SMAD2/SMAD3 for downstream signaling. As shown in Table 4 and Fig. 5, the abundance of SMAD2/SMAD3 were detected and validated, which supports the active involvement of activins and inhibins.

Among the TGF-beta superfamily ligands, bone morphogenetic proteins (BMPs) are believed to be very important in follicular development^62^. In present study, the expression profiles of BMP2, BMP4 and BMP6 were investigated. Interestingly, the expression of *BMPs* is consistently low across the developmental stages. As shown in Table 4, the FPKM value of *BMP2* varied from 2.5 (6 mm follicles) to 0.9 (F1 follicles) implying that they may not play a substantial role in follicular development. *BMP4*, which can reduce *FSHR* expression and promote the expression of *StAR*, *CYP11A* and *AMH* thus coordinating the follicular growth^63^, also shared a similar expression profile. Its FPKM value is similar in 6 mm and F1 follicles, at 40.5 and 38.9 respectively. In contrast to *BMP2* and *BMP4*, *BMP6* is reported to stimulate *FSHR* and *LHR* expression and promote differentiation of granulosa cells^64^. In present study, *BMP6* also showed a low FPKM value, varying from 3.8 (6 mm follicles) to 0.1 (F1 follicles). The BMPs bind their receptor type II (BMPRII) which activates SMAD5 for downstream signaling^62^. In present study, *BMPRII* transcript is found to be consistent across different stages, with FPKM varying only slightly from 76.5 (F1 follicles), 96.8 (F5 follicles) to 106.7 (6 mm follicles). This finding, together with the high abundance of *SMAD5* with FPKM varied from 70.9 (6 mm follicles), 176.5 (F5 follicles) to 202.4 (F1 follicles), hinted that BMPs may also have sources other than granulosa cells, for instance, neighboring cells. In small follicles, BMPs such as BMP15 were reported to be secreted from the oocytes to promote proliferation of granulosa cells^65^. In large follicles, owing to the limit of simple diffusion, BMPs may have come from theca cells which are in closer proximity of granulosa cells. To add to this, *BMP6* was reported to be expressed in abundance in theca cells and able to influence the granulosa cell proliferation^64^. In fact, signaling molecules such as BMPs from mesodermal origin cells have been proposed to play key roles in guiding the differentiation of endodermal origin cells such as granulosa cells^66^.

TGF-beta family members activate the Inhibitor of DNA-binding/differentiation proteins (IDs) in a SMAD-dependent manner thus connecting their role with these ID members^67^. As transcriptional regulators, IDs negatively regulate a wide range of genes with E boxes and are implicated in the regulation of cell fate^68^. In present study, multiple ID genes were identified in both chicken and bovine granulosa cell expression profiles, emphasizing their conserved roles along vertebrate follicular development. Our gene expression analyses further validated that ID1, ID2 and ID3 transcripts showed their highest expression level in F5 follicles (Fig. 5E). Such expression profiles are different from previous report that ID1 and ID3 transcripts showed the down-regulation and ID2 transcript showed up-regulation during the follicular development^69^. The discrepancy may result from the difference in sample numbers (n = 8 in present study) and reference genes (GAPDH and EF1A in present study). The prehierarchical follicles are normally maintained in an undifferentiated state by inhibitory MAP kinase (MAPK) signaling pathway mediated by epidermal growth factor ligands (EGFRLs)^70^. The high expression of ID1, ID3 and ID4 detected in small follicles were suggested to inhibit MAPK/EGFLs expression^71^. In addition, the up-regulation of ID2 protein along follicular growth was suggested to be responsible for the up-regulation of FSHR expression, a key step in follicular selection or recruitment^5,69^. The distinct expression profile of IDs revealed in our study strongly suggests the necessity of tracing the developmental follicles in real time in an effort to reveal the mechanisms of follicular selection.

In summary, high-throughput transcriptome analyses were employed to study the expression profiles of granulosa cells in follicles of three developmental stages in chicken. The analyses elucidated a clear tendency of granulosa cells in shifting its expression profile from proliferation to differentiation during follicular development. Transcripts down-regulated during this process were mainly associated with cell division, cell cycle and DNA replication while the up-regulated transcripts were related to ribosomal function, lipid metabolism and protein synthesis. Both chicken and bovine follicles showed a similar expression profile in steroidogenesis and cytoskeleton adjustment in follicular development. Our study for the first time provided the complete gene expression profiles along chicken follicular development, supporting the active involvement of many genes characterized in cell signaling (*AMH*,* Inhibins*, *Activins*, *BMPs*) and transcription factors (*SMAD3*,* SMAD5*, *ID1*, *ID2*, *ID3*). Their temporal expression profiles supports the notion of continual cross-talk between granulosa cells and its neighboring cells and shed light on the mechanisms behind avian follicular selection and pave the way to the better understanding of reproductive efficiency.

## Supplementary information

supplementary information
