# Supplementary material for: Transcriptomic Diversification of Granulosa Cells during Follicular Development in Chicken
Source: Sci Rep. 2019 Apr 2;9:5462. doi: 10.1038/s41598-019-41132-1 (PMC6445143; doi:10.1038/s41598-019-41132-1)
Supplement: Supplementary file 1 — supplementary information [file 41598_2019_41132_MOESM1_ESM.pdf]

Transcriptomic Diversification of Granulosa Cells during Follicular Development in Chicken

Guoqiang ZHU, Chao FANG, Jing Li, Chunheng MO, Yajun WANG, Juan LI*

(Key Laboratory of Bio-resources and Eco-environment of Ministry of Education, College of Life Sciences, Sichuan University, Chengdu 610065, PR China)

Keywords: Chicken, ovary, follicular development, granulosa cell, transcriptome analysis

Running title: Transcriptome analysis of chicken ovarian follicles

* Correspondence to:

Prof. Juan Li

Key Laboratory of Bio-resources and Eco-environment of Ministry of Education,

College of Life Sciences, Sichuan University, Chengdu, 610064, PR China

Email: lijuanscuhk@163.com Tel: 86-28-85415025

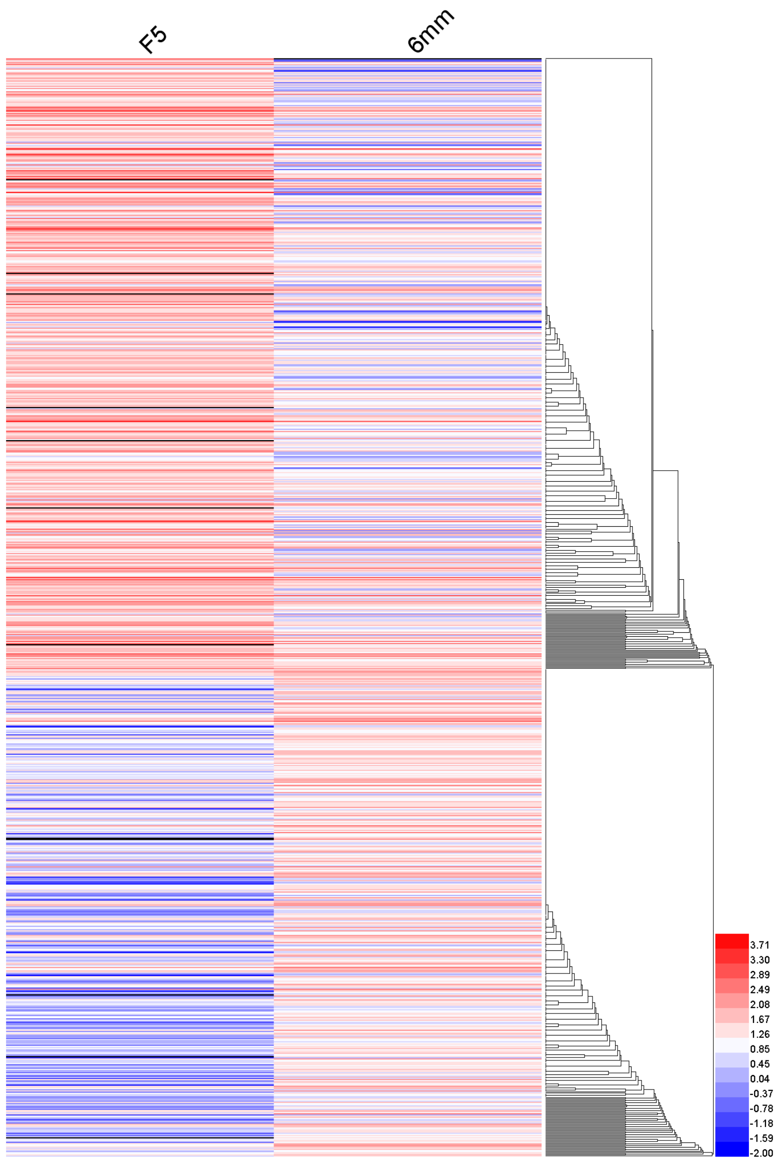

Fig. S1

The heatmap displaying the gene expression profiles between chicken F5 and 6 mm follicles.

The FPKM were directly from the chicken RNA-seq data (NO:GSE112470).

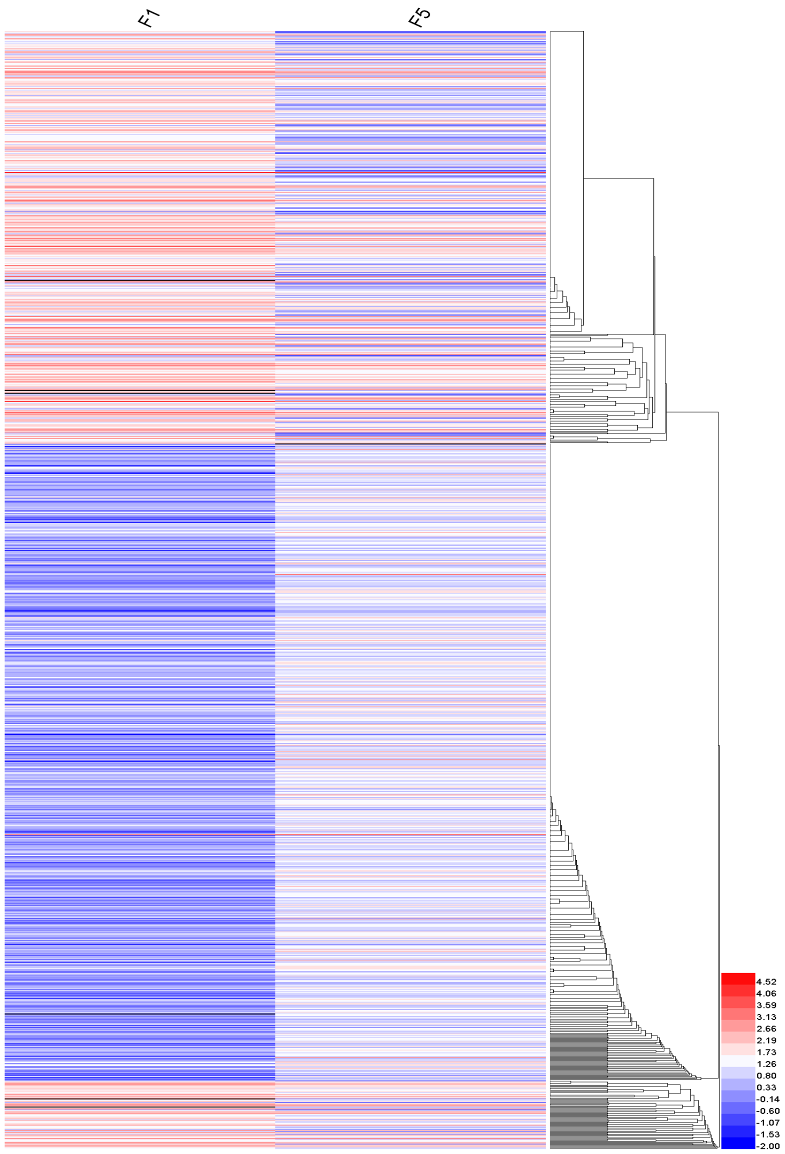

Fig. S2

The heatmap displaying the gene expression profiles between chicken F1 and F5 follicles. The FPKM were directly from the chicken RNA-seq data (NO:GSE112470)

Table S1: The FPKM value of 82 genes in common during follicle development in both chicken and bovine

Gene Name	chicken-6mm	chicken-F1	bovine-3.2mm	bovine-15.3mm
Steroid synthesis				
**NR5A2**	0.3	143.4	377.4	2083.8
**RASD1**	1.6	661.4	45.6	107.6
**STAR**	2.1	599.2	29.7	98.7
**CYP11A1**	2.7	568.0	837.5	4871.0
**CYP51A1**	30.8	137.8	448.8	1305.2
**SCARB1**	7.6	24.2	526.4	1351.2
**LHCGR**	9.6	471.5	37.3	332.0
**CYP21A2**	12.4	1.0	85.6	39.4
Intracellular protein synthesis, transportation and enzymes				
**SORBS2**	0.8	29.7	230.7	608.9
**HERPUD1**	34.9	196.8	989.1	3385.1
**SQLE**	19.4	106.7	689.8	1752.3
**SEC61A1**	36.2	65.5	572.1	1305.2
**SSR1**	103.0	151.6	30.1	87.4
**QSOX1**	100.3	156.9	130.7	453.5
**TRAM2**	56.6	13.8	94.4	38.1
**AOAH**	14.2	0.5	390.7	152.2
**DCTD**	17.4	0.7	148.1	67.4
**EGLN3**	8.5	0.1	36.5	17.1
Intracellular signaling proteins				
**GRK5**	0.1	8.6	38.6	215.3
**SNX17**	26.7	115.4	340.1	687.4
**ARHGAP18**	5.3	17.2	36.3	762.7
**APC**	5.0	15.3	42.8	123.6
**OBSL1**	3.1	20.3	91.1	530.1
**GADD45B**	3.1	20.1	324.0	2233.4
**RNF34**	11.8	24.8	229.1	461.4
**PDP1**	8.9	27.0	237.2	762.7
**CHST2**	5.7	15.9	15.5	37.4
**GRAMD4**	3.1	8.6	190.0	469.5
**PTP4A2**	68.4	160.5	1278.3	3956.5
**STT3B**	57.2	112.8	118.6	349.7
**AHCYL2**	34.8	53.2	132.5	388.0
**PSAP**	444.9	556.7	126.2	469.5
**RAC3**	42.2	12.5	219.8	73.5
**APCDD1**	10.4	0.3	171.3	80.2
**CARHSP1**	56.7	9.3	272.5	105.8

**RGS5**	4.8	0.0	57.3	27.9
**IQCA1**	6.1	0.0	49.9	23.0
**DNAJC6**	27.1	3.4	69.1	32.0
**HOMER2**	9.4	0.4	163.1	67.4
**MTSS1**	56.6	16.6	714.1	326.3
**SMOC2**	11.0	0.1	652.6	315.2
Cytoskeleton constituents and the extracellular matrix				
**CDC42EP4**	19.1	79.6	247.3	675.6
**SPTAN1**	29.4	49.1	744.4	1579.2
**FLNB**	131.0	198.6	134.4	309.8
**TEX264**	28.6	51.0	47.8	113.4
**EPB41**	6.4	1.0	46.5	19.4
**CDH3**	181.0	9.5	177.3	62.9
**ACTA1**	91.5	3.6	335.5	98.7
**FHL2**	17.3	0.1	372.2	117.4
**EMCN**	52.6	0.3	48.2	23.8
**COL6A1**	27.7	0.0	288.0	105.8
Transmembrane transporters, channel proteins and receptors				
**SLCO3A1**	1.1	35.1	183.5	415.9
**SEMA6A**	13.9	45.4	35.3	97.0
**SLC17A5**	9.1	21.2	247.3	711.6
**ABCA3**	45.1	57.8	124.5	243.0
**FAM174B**	20.0	38.9	44.9	152.2
**ST6GAL1**	5.3	0.5	72.5	28.3
**GPC4**	4.1	0.3	288.0	105.8
**CLIC2**	4.8	0.0	53.8	26.4
**ENTPD1**	5.2	0.1	99.0	46.9
**FXYD6**	10.0	0.0	982.3	309.8
**AQP1**	36.4	0.0	172.4	53.8
**RBP3**	4.5	0.0	46.9	23.0
**PECAM1**	12.9	2.3	88.0	40.1
Transcription factors				
**ID1**	31.3	75.2	28.1	60.8
**ID2**	191.3	262.1	467.9	2083.8
**ID3**	39.6	99.7	137.2	578.0
**GATA6**	118.9	234.4	2048.0	5595.3
**ZNF462**	6.0	18.6	91.1	207.9
**ZNF609**	32.2	50.9	94.4	343.7
**ANKRD50**	16.5	23.5	213.8	503.2
**MYCBP2**	11.1	17.1	166.6	568.1
**TGIF1**	20.7	3.6	362.0	102.2
**EMX2**	75.8	11.4	75.1	28.8
**TRIM2**	55.1	7.3	2320.1	687.4

**FOS**	40.1	4.5	146.0	36.1
**FOXP4**	103.2	7.1	1031.1	394.8
**MYC**	23.8	5.1	1499.2	92.1
Signaling molecules				
**IGFBP4**	356.7	1824.1	71.5	520.9
**INHBA**	1.1	53.3	4011.7	15554.0
**IGF2**	50.2	6.3	121.9	56.7
**BMP15**	18.3	0.2	94.4	46.0
